# Changing Climate and Overgrazing Are Decimating Mongolian Steppes

**DOI:** 10.1371/journal.pone.0057599

**Published:** 2013-02-25

**Authors:** Yi Y. Liu, Jason P. Evans, Matthew F. McCabe, Richard A. M. de Jeu, Albert I. J. M. van Dijk, Albertus J. Dolman, Izuru Saizen

**Affiliations:** 1 Water Research Centre, School of Civil and Environmental Engineering, University of New South Wales, Sydney, Australia; 2 Climate Change Research Centre, University of New South Wales, Sydney, Australia; 3 Earth and Climate Cluster, Department of Earth Sciences, Faculty of Earth and Life Sciences, VU University Amsterdam, Amsterdam, The Netherlands; 4 CSIRO Land and Water, Black Mountain Laboratories, Canberra, Australia; 5 Water Desalination and Reuse Center, King Abdullah University of Science and Technology, Thuwal, Saudi Arabia; 6 Fenner School of Environment & Society, The Australian National University, Canberra, Australia; 7 Graduate School of Global Environmental Studies, Kyoto University, Kyoto, Japan; Lakehead University, Canada

## Abstract

Satellite observations identify the Mongolian steppes as a hotspot of global biomass reduction, the extent of which is comparable with tropical rainforest deforestation. To conserve or restore these grasslands, the relative contributions of climate and human activities to degradation need to be understood. Here we use a recently developed 21-year (1988–2008) record of satellite based vegetation optical depth (VOD, a proxy for vegetation water content and aboveground biomass), to show that nearly all steppe grasslands in Mongolia experienced significant decreases in VOD. Approximately 60% of the VOD declines can be directly explained by variations in rainfall and surface temperature. After removing these climate induced influences, a significant decreasing trend still persists in the VOD residuals across regions of Mongolia. Correlations in spatial patterns and temporal trends suggest that a marked increase in goat density with associated grazing pressures and wild fires are the most likely non-climatic factors behind grassland degradation.

## Introduction

**Figure 1:**
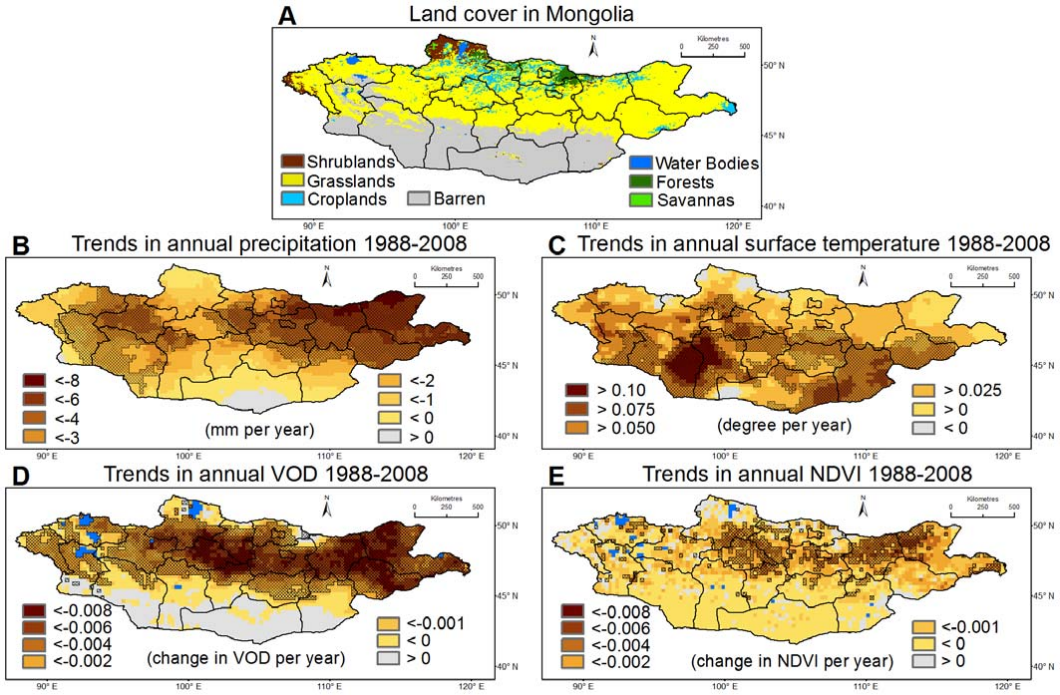
Land cover and trends in climatic variables and vegetation indices. (A) Land cover in Mongolia derived from the Moderate Resolution Spectroradiometer (MODIS) satellite data. (B) The trend in annual total precipitation using Mann-Kendall trend test. The areas with statistically significant trend (*P*<0.05) are hatched. (C, D and E) Same as (B), but for annual average surface temperature, annual average VOD and NDVI, respectively.

The extensive steppes of Mongolia are among the largest contiguous expanses of grassland in the world and encompass a region of considerable ecological importance both nationally and internationally. Here we present evidence that Mongolia's fragile steppe ecosystem is degrading at a rapid rate. Mongolia experiences an extreme continental climate characterized by long cold winters, short summers and low annual precipitation. Average temperature in Mongolia increased by more than 2 °C since 1940 and all seasons have become warmer, whereas annual total precipitation dropped by 7% during the same period [1]. In addition, following the privatization of livestock ownership in the early 1990s and rising international demand for cashmere products, the goat population increased from 4.4 million in 1988 to about 20 million in 2008 [2]. A changing climate and increased grazing pressure have intensified the threat of desert expansion from south Mongolia towards the central and northern grasslands (Figure 1A). In 2002, it was estimated that over 70% of the total territory was degraded relative to its natural state [3].

To assess the risk of future ecosystem change and the potential for remediation, the relative impacts of climate and human activities need to be understood. Here, we utilize a recently developed long term satellite based vegetation optical depth (VOD) dataset [4]–[7] coupled with in situ measurements to investigate climate and anthropogenic factors affecting the vegetative state of Mongolian grasslands. To aid in this analysis, gridded estimates of precipitation, surface temperature and a remote sensing based greenness index (Normalized Difference Vegetation Index, NDVI) were also used. All data were resampled to a common spatial resolution of 0.25° (about 25 km) to enable direct inter-comparison. Further details on these data can be found in the Materials and Methods section.

## Results and Discussion

Non-parametric Mann-Kendall trend tests [8] were applied to time series of annual precipitation, temperature, VOD and NDVI (VOD_AVG_ and NDVI_AVG_, respectively). Results of this analysis indicate that the majority of the country experienced decreasing precipitation and increasing temperature over the period 1988–2008, albeit with considerable spatial heterogeneity (Figure 1B and C). Nearly all steppe regions experienced significant declines in VOD_AVG_ during 1988 – 2008 (*p*<0.05, Figure 1D), broadly consistent with changes in rainfall and temperature. The steppe regions with significant VOD declines are the focus of this study. Large scale NDVI_AVG_ declines were also observed, but the extent exhibiting significant declines is smaller (Figure 1E). We attribute this to the different vegetation characteristics represented by VOD (which detects water content in both foliage and woody biomass) and NDVI (which is a measure of chlorophyll abundance in the leaves) [6], [9]. A change in the canopy does not inevitably mean that the vegetation biomass changes in proportion or in the same direction, and vice versa [10]. Where the total above ground biomass (represented by VOD) of grassland significantly declines with decreasing precipitation, the magnitude of NDVI reduction may be less provided if the change in the chlorophyll abundance of the leaves is small.

During the study period from 1988–2008, maximum monthly VOD values (VOD_MAX_) for each year generally peaked around July/August most of the time, and this occasionally occurs earlier or later in either June or September. The VOD_MAX_ values are highly correlated with VOD_AVG_, particularly over the grasslands (Figure S3) and long term trends in VOD_MAX_ are statistically the same as in VOD_AVG_ (Figure S4). Long term field studies in neighboring Chinese Inner Mongolia show that annual total above ground biomass is strongly and positively correlated with precipitation during the growth period, a few months prior to peak biomass [11]–[12]. Conversely, surface temperature may have negative impacts on the biomass production [13]–[14]. To examine this, we performed regressions between varying periods of precipitation and temperature and VOD_MAX_ for each grid cell [15] in order to predict VOD (VOD_EST_; see Materials and Methods).

**Figure 2:**
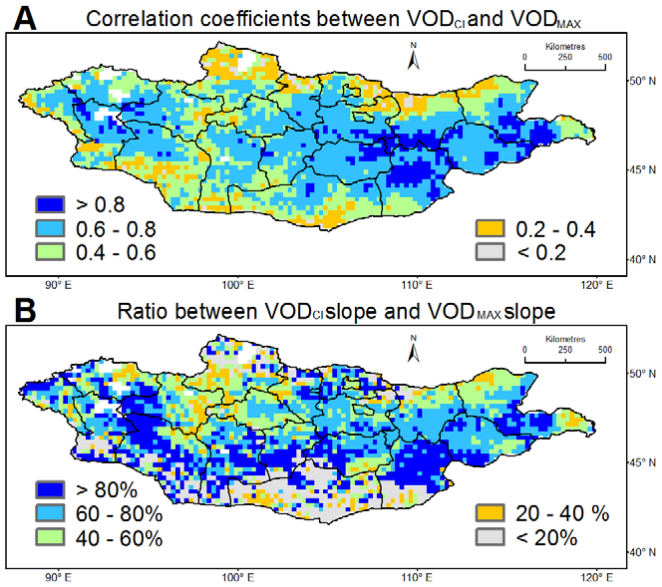
Relationship between VOD_CI_ and VOD_MAX_. (A) Correlation coefficients (r^2^) between VOD_CI_ and satellite-based VOD_MAX_ during 1988–2008. (B) Ratio (%) between the slope of VOD_CI_ and the slope of satellite-based VOD_MAX_ for the period 1988–2008.

For each grid cell, the predicted VOD_EST_ showing the highest correlation with VOD_MAX_ was selected and interpreted as the “climate induced VOD” (VOD_CI_), representing the direct contribution of natural climate influences on VOD_MAX_. The resulting VOD_CI_ suggests that VOD_MAX_ is strongly influenced by precipitation and to a lesser extent temperature (see Figure S5) for about 0 to 5 months prior to peak biomass (see Figure S6 and S7), consistent with previous studies [11]–[12]. Based on our analysis, temperature has a negative impact on the VOD over the Mongolian grasslands and a positive impact on the VOD over the northern most area of Mongolia (>50°N). These results agree with previous observations which found that water is the main limiting factor for vegetation growth over south Mongolia, while energy is the major growth limiting factor over north Mongolia [16]–[17]. On average, about 60% of observed variance in VOD_MAX_ is statistically explained, with higher values (>0.60) over the west and the central-east to east (Figure 2A). VOD_CI_ also explains about 60% of VOD_MAX_ declines, increasing to more than 80% over western and southeastern Mongolia (Figure 2B). This confirms that both precipitation and temperature during the growth period play a dominant role in controlling interannual grassland production [12].

A residual vegetation optical depth (VOD_RES_) was calculated for each grid cell and year by subtracting VOD_CI_ from the observed VOD_MAX_ (Figure S8), with the expectation that this residual VOD contains information on influences other than climate, including human induced and other ecological disturbances.

**Figure 3:**
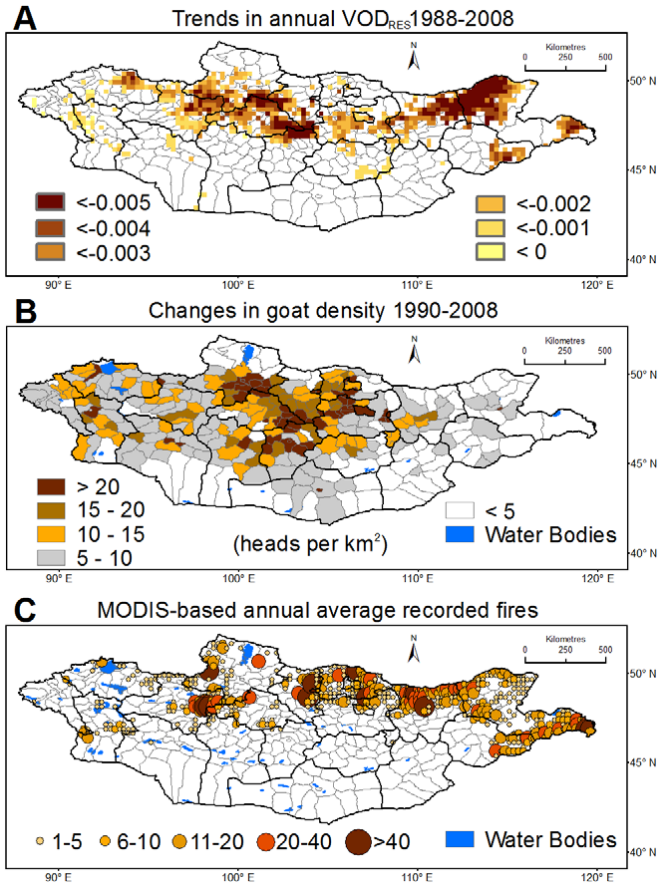
Trends in VOD residuals, changes in goat density and fire hotspots. (A) Trends in VOD residuals after removing the influence of climatic factors. Only statistically significant trends (*p*<0.05) are shown. The analysis conducted on VOD was also applied on NDVI. No significant trends were observed in NDVI residuals and thus they are not shown here. (B) Differences in goat density (heads per square kilometer) between 2008 and 1990. (C) Annual average number of recorded fires for each 0.25° grid cell between 2001 and 2008, based on MODIS global monthly fire location product.

VOD_RES_ shows significant (*p*<0.05) declining trends between 1988 and 2008 (Figure 3A). During this time, central Mongolia experienced considerable goat density increases (Figure 3B); goat numbers for 2002–2008 were about four and two times those of 1988–1994 and 1995–2001, respectively [18]. The spatial and also temporal agreement in trend patterns (see Figure S9) suggests that the rapid increase in goat numbers may very well have contributed to the significant declines in VOD_RES_ over central Mongolia, as well as across the central-eastern and western regions.

To identify plausible causes for the biomass decrease over east Mongolia, we examine the time series of VOD_RES_ in greater detail. VOD_RES_ values since 1996 are well below the long term average (see Figure S10), and coincide with a period of increased biomass burning. During 1996–1998, approximately 210,000 km^2^ of land in eastern Mongolia was burnt, representing a fivefold increase compared with the previous decade [19]. Between 2001 and 2008, large scale fires took place almost every year [20] (Figure 3C). Continuous fire events during the growing season have severe consequences for the annual peak VOD (see Figure S11 for one example). Field experiments in eastern Mongolia showed that consecutive fire events resulted in a stronger decrease of vegetation cover than grazing [21]. The spatial and temporal agreement for these regions suggests that the VOD_RES_ declines are primarily attributable to burning [22].

## Conclusions

We present evidence that: (1) almost the entire Mongolian steppe region experienced significant vegetation biomass declines between 1988 and 2008; (2) about 60% of the decline can be attributed to climate trends: in particular, decreasing precipitation and increasing temperature; and (3) the dramatic increase in goat numbers and grassland burning is likely to account for most of the remaining decline. Average air temperature is projected to increase and precipitation to decrease in Mongolia over the next three decades [23]. The implications of this changing climate will further stress an already fragile environment and likely accelerate grassland degradation. There is an urgent need to develop and implement effective strategies for sustainable grazing practices, and reduce the incidence and severity of burning in order to improve the resilience of the Mongolian steppes. Understanding the competing influences of climate, land management and global demand for a niche agricultural product like cashmere will be key to protecting these ecosystems from further degradation.

## Materials and Methods

### Vegetation Optical Depth (VOD) and Normalized Difference Vegetation Index (NDVI)

The VOD retrievals used in this study are derived using the Land Parameter Retrieval Model, developed by the VU University Amsterdam in collaboration with National Aeronautics and Space Administration [4]–[5], [24]. In retrieving the VOD, the land surface temperature is estimated from the Ka-band brightness temperature, a wavelength band common to all successive sensors used here [25]. The vegetation optical depth and soil dielectric constant are derived simultaneously [4], and the soil moisture is subsequently retrieved from the dielectric constant using the Wang-Schmugge model [26].

The VOD is a dimensionless parameter that can be interpreted as being directly proportional to vegetation water content, mainly varying with wavelength of the sensor, vegetation structure and viewing angle [4], [24], [27]–[29]. As a result, values of VOD derived from different instruments may differ. However, they are highly correlated, which creates the opportunity to rescale VOD values from different instruments against one reference and merge into one time series. The VOD product used in this study was derived by merging VOD datasets from two independent passive microwave missions: the Special Sensor Microwave Imager (1987–2007) and the Advanced Microwave Scanning Radiometer for EOS (2002–2008), through use of the cumulative distribution function (CDF) matching technique [6]. The merged long term VOD dataset can be used monitor global changes in total aboveground vegetation water content and biomass over various land cover types, including grassland, shrubland, cropland, boreal forest and tropical forest [8].

The NDVI 3 g data (1981–2010), derived from the AVHRR instruments onboard the NOAA satellite series was available from the Global Inventory Monitoring and Modeling Studies (GIMMS). VOD retrievals are only available when surface temperature is above 0° (typically from middle April to middle October). Since the analysis of this study was conducted at the monthly interval, we only used the VOD data from May through September. The approach to distinguish climate and human induced contribution on the interannual VOD variations is based on the month with the maximum VOD value (VOD_MAX_) which is normally observed between June and September, and therefore the data gap in the winter time has little influence on the results of this study.

When VOD retrievals are not available, concurrent NDVI data were also masked out. For a spatially equivalent comparison, VOD and NDVI were both resampled to 0.25°. Both products capture similar spatial patterns of vegetation distribution and seasonal cycles (Figure S1 and 2).

### Land cover product

The land cover data followed the International Geosphere Biosphere Programme (IGBP) scheme [30] and were derived from Moderate Resolution Spectroradiometer (MODIS) satellite data (MCD12Q1, ftp://e4ftl01.cr.usgs.gov/MOTA/MCD12Q1.005/2001.01.01) at 500 m spatial resolution.

### Precipitation and temperature datasets

To analyse precipitation and temperature based climate changes over this region, averages of three 0.5° gridded monthly precipitation products (Global Precipitation Climatology Centre (GPCC) [31], University of Delaware (UDel) [32], Climatic Research Unit (CRU) at the University of East Anglia [33]) and two surface temperature products (UDel [32] and CRU [33]) were used from 1988 through 2008, to avoid any potential bias existing in an individual product. These data were interpolated into 0.25° spatial fields to correspond with the satellite based VOD retrievals.

### Monthly fire location product

The Global Monthly Fire Location Product (MCD14ML) contains the geographic location (latitude and longitude), date (at the daily interval) and detection confidence (0–100%) for fires detected by the Terra and Aqua MODIS combined. Only the fires with 100% detection confidence from 2001 through 2008 were used in this study. Detections were accumulated for each 0.25° grid cell to derive the number of wild land fires per year for direct comparison with the 0.25° VOD product. For instance, two fires which were detected within the same 0.25° grid cell on the same day were counted as two wild land fires, as the influences of these two fires combined on the VOD observations were expected to be stronger than only one fire.

### Statistical methods

Spearman's rank correlation coefficient was calculated to quantify the relationship between two variables (e.g. VOD_CI_ and VOD_MAX_). The Mann-Kendall trend test was applied to identify the direction and significance of long term time series. Both techniques are non-parametric, based on relative ranks of sample data rather than absolute values, and do not require any assumptions about the nature of the relationship, other than being monotonic.

### Distinguishing climatic and non-climatic factors

We performed multiple linear regressions between varying periods of precipitation and temperature and VOD_MAX_ for each grid cell in order to predict an expected VOD_EST_. 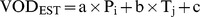



where VOD_EST_ is the estimated VOD resulting from precipitation and temperature influences and P_i_ and T_j_ represent precipitation totals (unit: mm) and temperature averages (unit: K) for accumulation periods of between 1 to 8 months, and for lead times of 0 to 7 months prior to the time of VOD_MAX_. As the peak precipitation normally occurs between June and August, the peak biomass is expected to be between June and September. Therefore the selection of the month with maximum VOD value is limited to the period from June and September. For instance, if VOD_MAX_ is reached in August, then precipitation with a 7-month accumulation period and 1-month lead time represents the total precipitation amount from January through July. P_i_ and T_j_ are not necessarily for the same accumulation periods and lead times. For each grid cell, the predicted VOD_EST_ showing the highest correlation with VOD_MAX_ was selected and identified as the “climate induced VOD” (VOD_CI_).

### Impacts of repeated fire events

We show a case study that compares the VOD values in eastern Mongolia for two years with similar precipitation patterns (see Figure S11). The most distinct difference between these two years is that one year had many fire events and the other few. In the year with few fire events (2001), VOD values apparently increased from May through August with precipitation. In the year with repeated fire events (2007), VOD values apparently declined from May to June, even though the precipitation is comparable with 2001. The most likely reason for the VOD decline is the fire events in June. The VOD value in August 2007 went up with reduced fire events and increased precipitation, but its value was only 80% of August 2001. This case study demonstrates the severe influence of frequent fire events on the annual peak value and seasonal cycle of VOD.

## Supporting Information

Figure S1 **Spatial patterns of VOD and NDVI.** Annual average satellite observed (A) VOD and (B) NDVI for the period 1988–2008. The blue grid cells stand for open water bodies.
(TIF)

Figure S2 **Relationship between VOD and NDVI seasonality.** Spearman's ranked correlation coefficients (r) between VOD and NDVI seasonality derived from their overlapping period 1988 – 2008.
(TIF)

Figure S3 **Relationship between VOD_AVG_ and VOD_MAX_.** Spearman's ranked correlation coefficients (r) between annual average VOD (VOD_AVG_) and annual maximum monthly VOD (VOD_MAX_) during 1988 – 2008.
(TIF)

Figure S4 **Long term trends in VOD_MAX_ over 1988-2008.** Trends in annual maximum monthly VOD (VOD_MAX_) (change per year) during 1988 – 2008 using non-parametric Mann-Kendall trend test. The areas with statistically significant (*p*<0.05) trend are hatched.
(TIF)

Figure S5 **Relationship between different VOD_CI_ estimates and VOD_MAX_.** Correlation coefficients (r^2^) (A) between precipitation-and-temperature-based VOD_CI_ and satellite-based VOD_MAX_ and (B) between precipitation-only-based VOD_CI_ and satellite-based VOD_MAX_ during 1988–2008. (C) Difference between (A) and (B), i.e. A minus B.
(TIF)

Figure S6 **Leading time and accumulation period of precipitation for VOD_CI_.** The (A) lead time (month) and (B) accumulation period (month) of precipitation corresponding to the climate induced vegetation optical depth (VOD_CI_).
(TIF)

Figure S7 **Leading time and accumulation period of temperature for VOD_CI_.** The (A) lead time (month) and (B) accumulation period (month) of temperature corresponding to the climate induced vegetation optical depth (VOD_CI_).
(TIF)

Figure S8 **Demonstration of deriving VOD_RES_ from VOD_MAX_ and VOD_CI_.** Example illustrating (A) the observed annual maximum VOD and optimal climate-induced VOD, and (B) their difference (i.e. observed minus climate-induced), and the slope and significance level of VOD_RES_ for the grid cell centered at 47.375°N and 103.125°E.
(TIF)

Figure S9 **VOD_RES_ and goat population.** Relationship between VOD_RES_ and goat population (mean ± standard deviation) over central Mongolia (including Arkhangai, Khovsgol and Bulgan) for 1988–1994, 1995–2001 and 2002–2008, respectively.
(TIF)

Figure S10 **Plot of VOD_RES_ over northeast Mongolia from 1988 through 2008.**
(TIF)

Figure S11 **Case study demonstrating the influence of continuous fire events on annual peak VOD value over northeast Mongolia.** (A) Monthly average VOD, rainfall and MODIS-based fire records for the year 2001. The number over the fire records bar indicates the total fire events observed by MODIS. (B) Same as (A), but for the year 2007.
(TIF)
