# Supplementary figures and images for: Changing Climate and Overgrazing Are Decimating Mongolian Steppes
Source: PLoS One. 2013 Feb 25;8(2):e57599. doi: 10.1371/journal.pone.0057599 (PMC3581472; doi:10.1371/journal.pone.0057599)

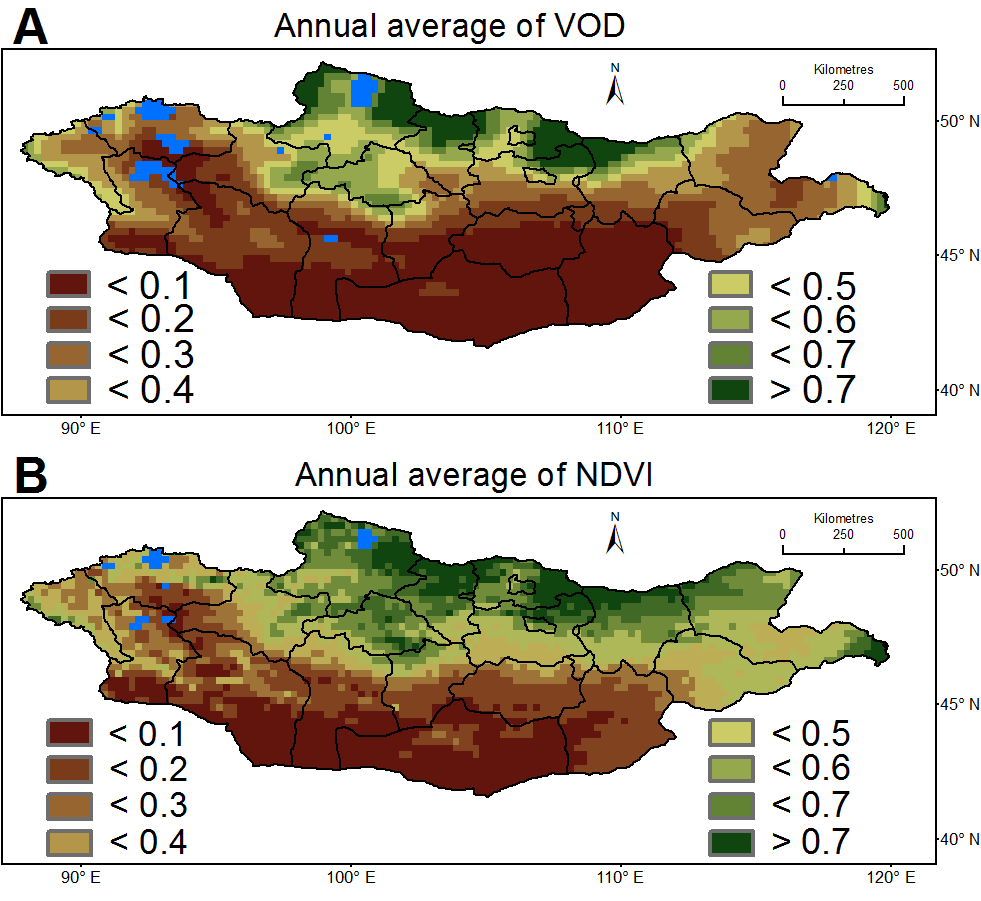

Supplement: Figure S1 — Spatial patterns of VOD and NDVI. Annual average satellite observed (A) VOD and (B) NDVI for the period 1988–2008. The blue grid cells stand for open water bodies. (TIF) [file pone.0057599.s001.tif]

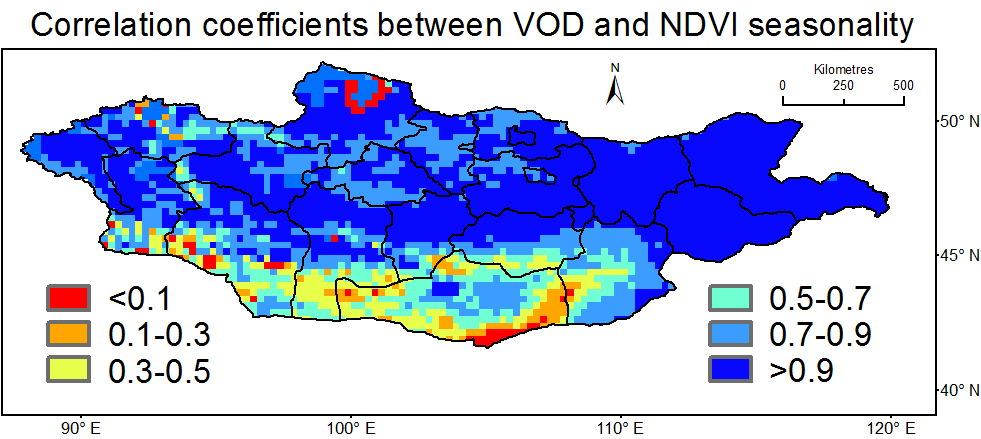

Supplement: Figure S2 — Relationship between VOD and NDVI seasonality. Spearman's ranked correlation coefficients (r) between VOD and NDVI seasonality derived from their overlapping period 1988 – 2008. (TIF) [file pone.0057599.s002.tif]

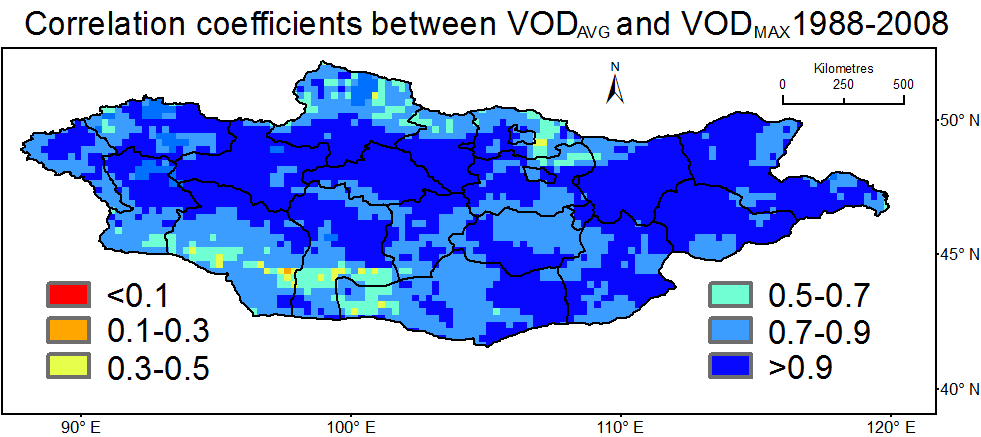

Supplement: Figure S3 — Relationship between VODAVG and VODMAX. Spearman's ranked correlation coefficients (r) between annual average VOD (VODAVG) and annual maximum monthly VOD (VODMAX) during 1988 – 2008. (TIF) [file pone.0057599.s003.tif]

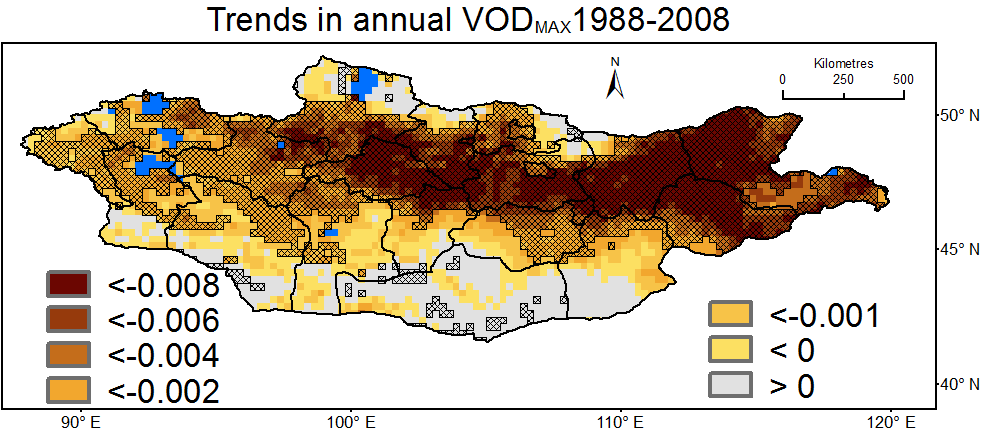

Supplement: Figure S4 — Long term trends in VODMAX over 1988-2008. Trends in annual maximum monthly VOD (VODMAX) (change per year) during 1988 – 2008 using non-parametric Mann-Kendall trend test. The areas with statistically significant (p<0.05) trend are hatched. (TIF) [file pone.0057599.s004.tif]

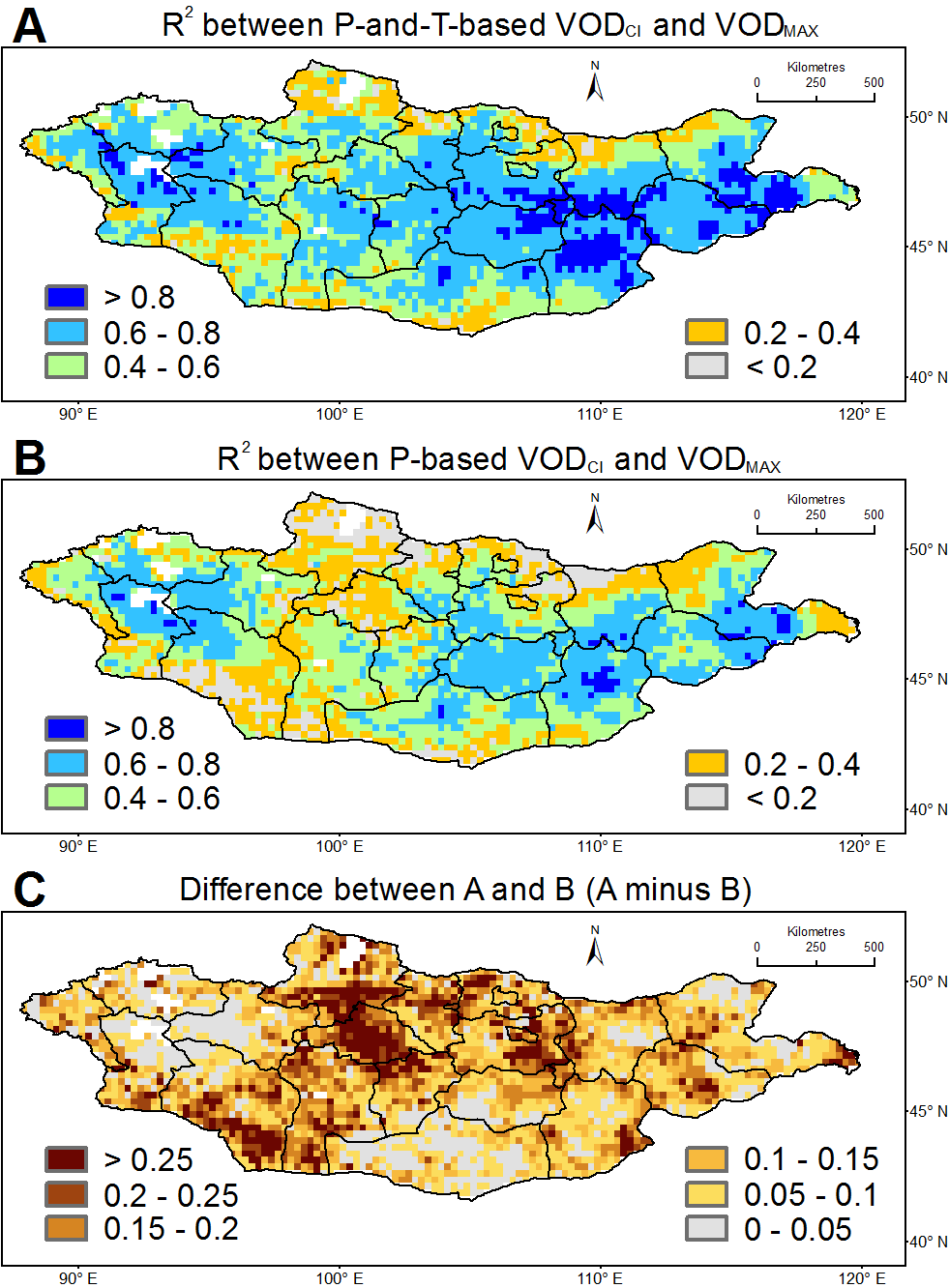

Supplement: Figure S5 — Relationship between different VODCI estimates and VODMAX. Correlation coefficients (r2) (A) between precipitation-and-temperature-based VODCI and satellite-based VODMAX and (B) between precipitation-only-based VODCI and satellite-based VODMAX during 1988–2008. (C) Difference between (A) and (B), i.e. A minus B. (TIF) [file pone.0057599.s005.tif]

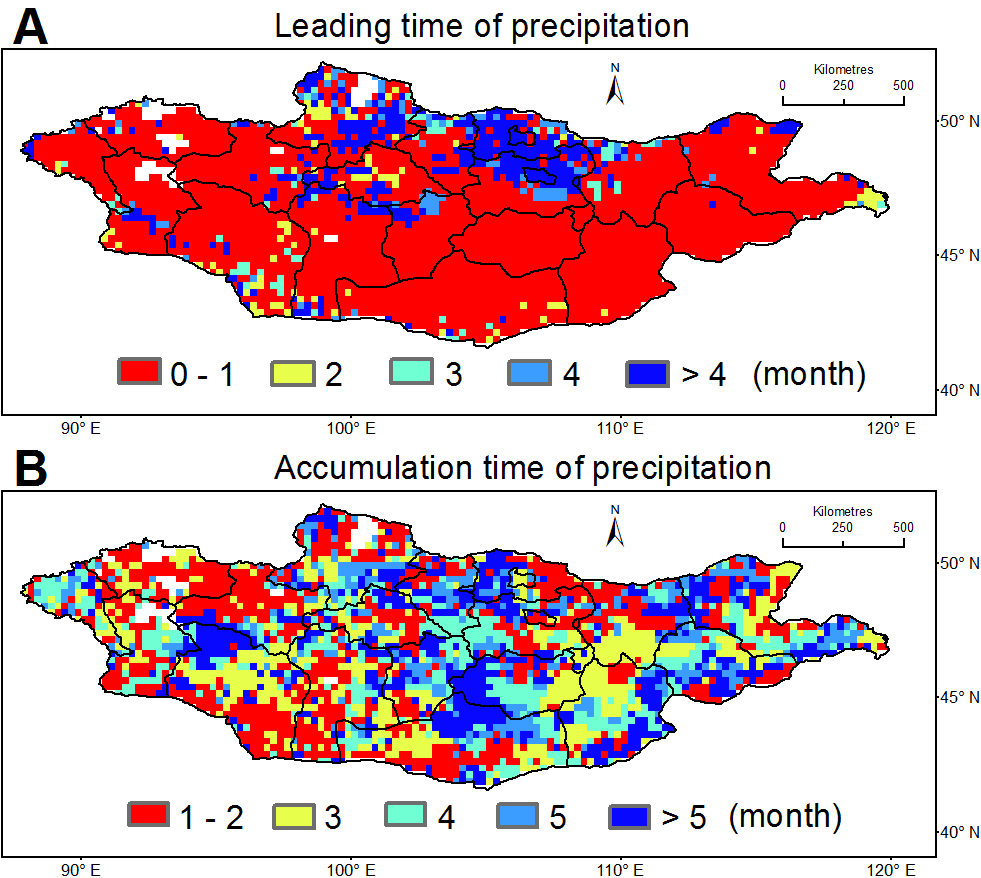

Supplement: Figure S6 — Leading time and accumulation period of precipitation for VODCI. The (A) lead time (month) and (B) accumulation period (month) of precipitation corresponding to the climate induced vegetation optical depth (VODCI). (TIF) [file pone.0057599.s006.tif]

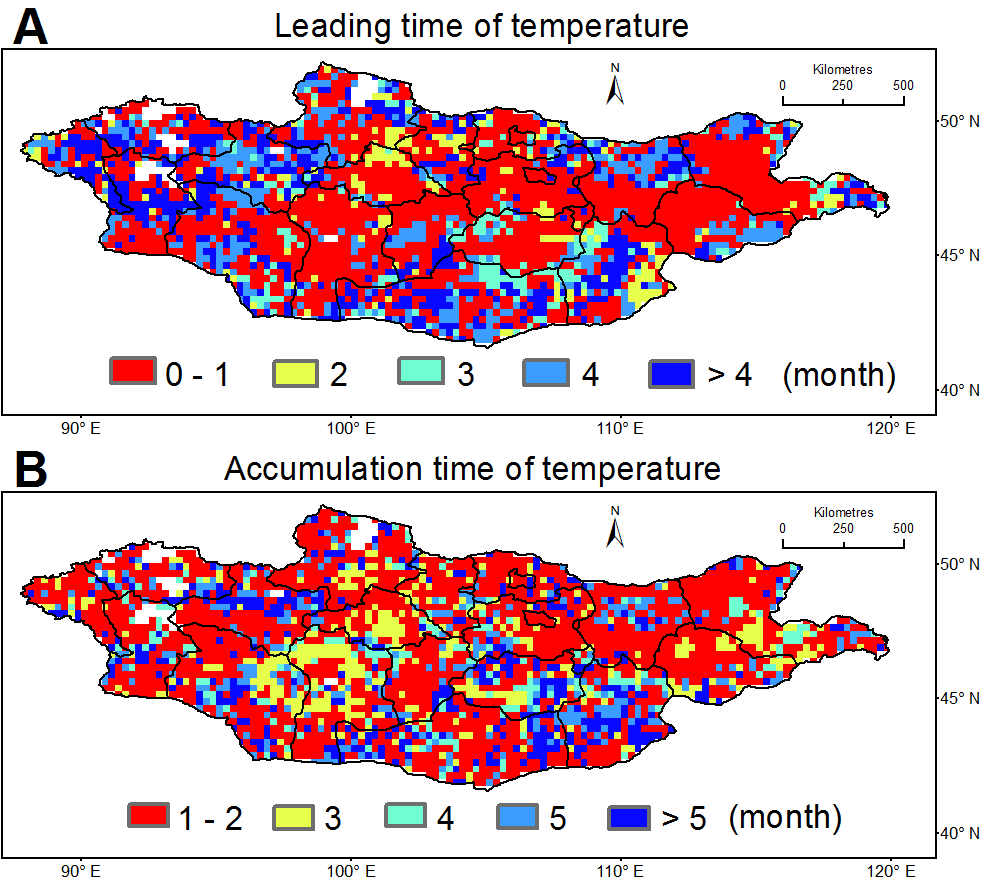

Supplement: Figure S7 — Leading time and accumulation period of temperature for VODCI. The (A) lead time (month) and (B) accumulation period (month) of temperature corresponding to the climate induced vegetation optical depth (VODCI). (TIF) [file pone.0057599.s007.tif]

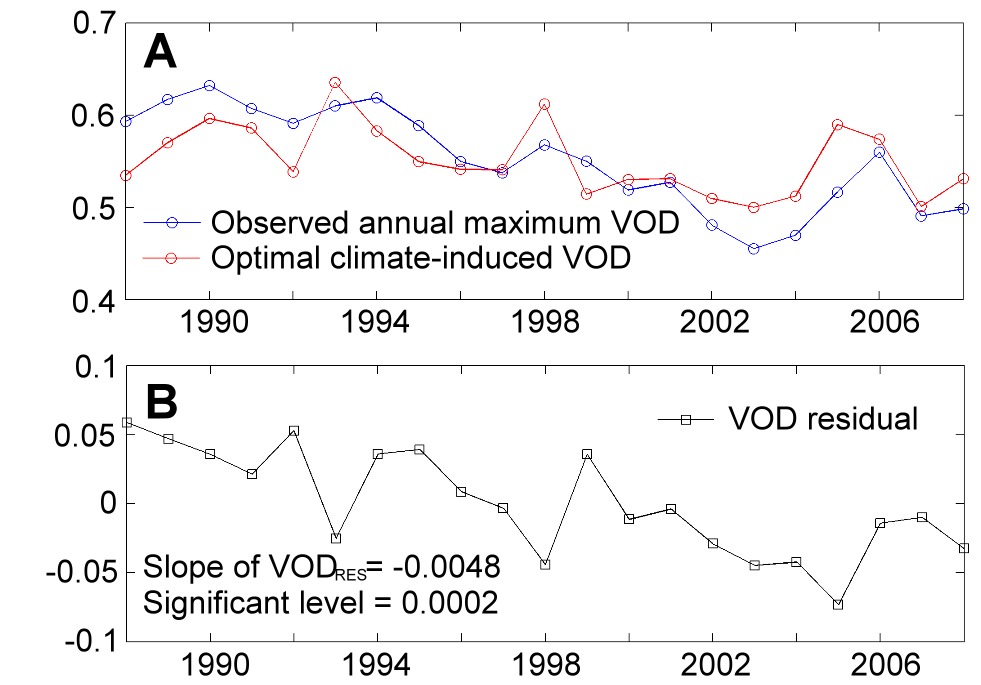

Supplement: Figure S8 — Demonstration of deriving VODRES from VODMAX and VODCI. Example illustrating (A) the observed annual maximum VOD and optimal climate-induced VOD, and (B) their difference (i.e. observed minus climate-induced), and the slope and significance level of VODRES for the grid cell centered at 47.375°N and 103.125°E. (TIF) [file pone.0057599.s008.tif]

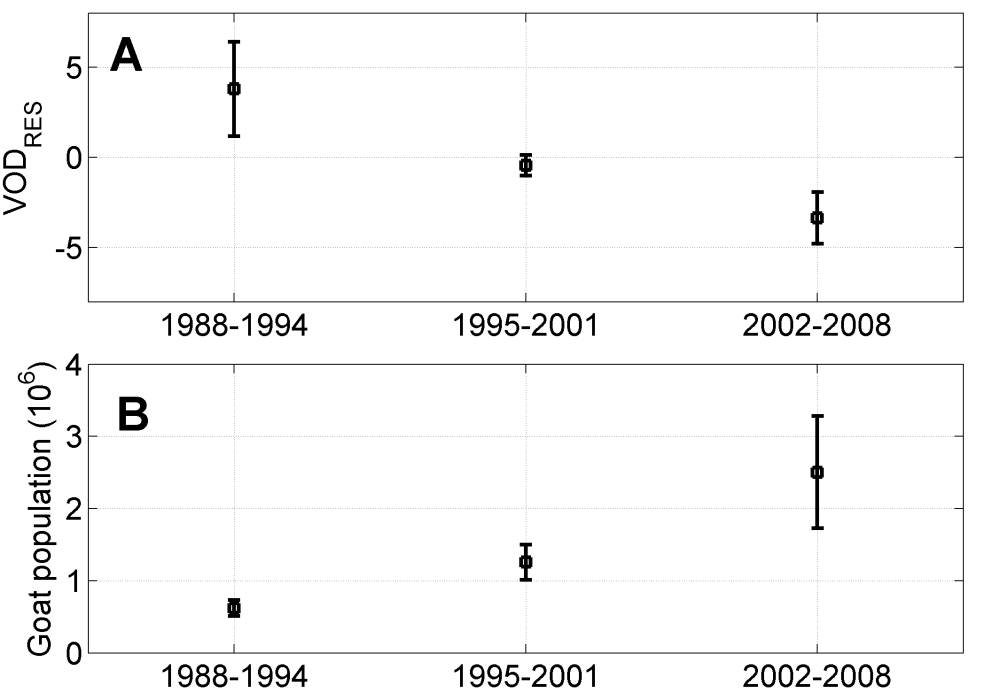

Supplement: Figure S9 — VODRES and goat population. Relationship between VODRES and goat population (mean ± standard deviation) over central Mongolia (including Arkhangai, Khovsgol and Bulgan) for 1988–1994, 1995–2001 and 2002–2008, respectively. (TIF) [file pone.0057599.s009.tif]

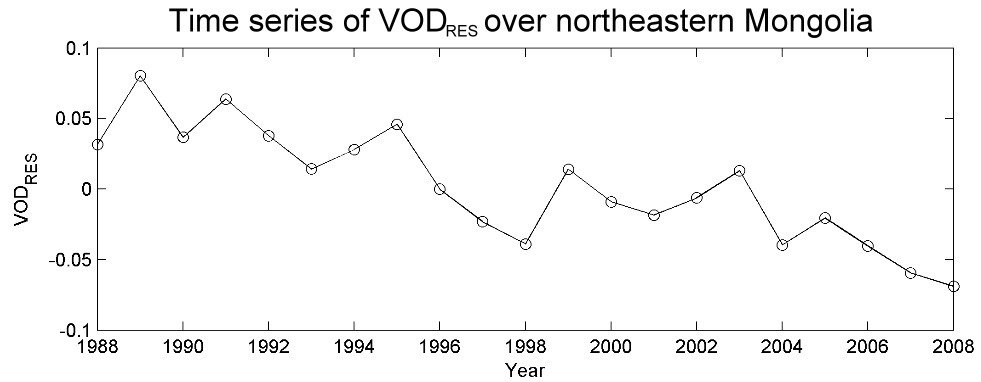

Supplement: Figure S10 — Plot of VODRES over northeast Mongolia from 1988 through 2008. (TIF) [file pone.0057599.s010.tif]

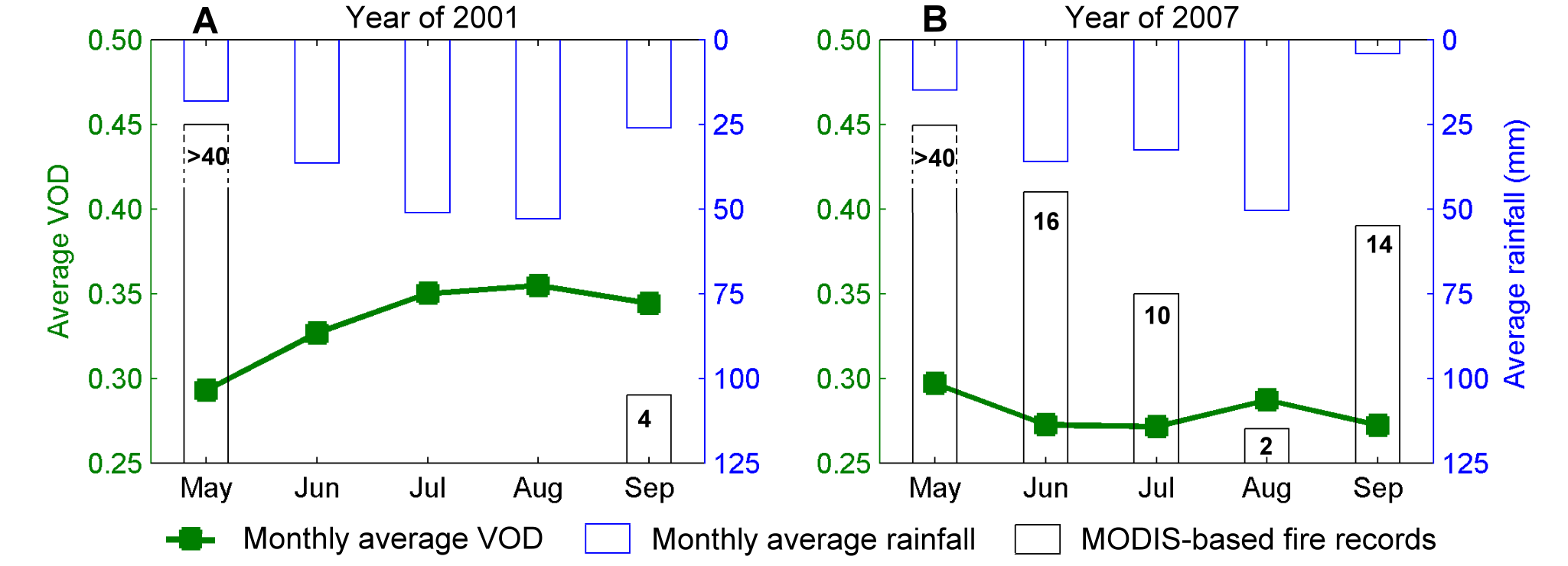

Supplement: Figure S11 — Case study demonstrating the influence of continuous fire events on annual peak VOD value over northeast Mongolia. (A) Monthly average VOD, rainfall and MODIS-based fire records for the year 2001. The number over the fire records bar indicates the total fire events observed by MODIS. (B) Same as (A), but for the year 2007. (TIF) [file pone.0057599.s011.tif]
